# Exploring population pharmacokinetic models in patients treated with vancomycin during continuous venovenous haemodiafiltration (CVVHDF)

**DOI:** 10.1186/s13054-021-03863-4

**Published:** 2021-12-20

**Authors:** Marcus Kirwan, Reema Munshi, Hannah O’Keeffe, Conor Judge, Mary Coyle, Evelyn Deasy, Yvelynne P. Kelly, Peter J. Lavin, Maria Donnelly, Deirdre M. D’Arcy

**Affiliations:** 1 School of Pharmacy and Pharmaceutical Sciences, Trinity College Dublin, Dublin 2, Ireland; 2 Department of Pharmacy, Tallaght University Hospital, Dublin 24, Ireland; 3 Department of Clinical Pharmacy, Umm Al-Qura University, Makkah, Saudi Arabia; 4 Department of Nephrology, Tallaght University Hospital, Dublin 24, Ireland; 5 Department of Critical Care, Tallaght University Hospital, Dublin 24, Ireland

**Keywords:** Acute kidney injury, Continuous renal replacement therapy, Antibiotics, Pharmacokinetics, Therapeutic dose monitoring

## Abstract

**Background:**

Therapeutic antibiotic dose monitoring can be particularly challenging in septic patients requiring renal replacement therapy. Our aim was to conduct an exploratory population pharmacokinetic (PK) analysis on PK of vancomycin following intermittent infusion in critically ill patients receiving continuous venovenous haemodiafiltration (CVVHDF); focussing on the influence of dialysis-related covariates.

**Methods:**

This was a retrospective single-centre tertiary level intensive care unit (ICU) study, which included patients treated concurrently with vancomycin and CVVHDF between January 2015 and July 2016. We extracted clinical, laboratory and dialysis data from the electronic healthcare record (EHR), using strict inclusion criteria. A population PK analysis was conducted with a one-compartment model using the PMetrics population PK modelling package. A base structural model was developed, with further analyses including clinical and dialysis-related data to improve model prediction through covariate inclusion. The final selected model simulated patient concentrations using probability of target attainment (PTA) plots to investigate the probability of different dosing regimens achieving target therapeutic concentrations.

**Results:**

A total of 106 vancomycin dosing intervals (155 levels) in 24 patients were examined. An acceptable 1-compartment base model was produced (Plots of observed vs. population predicted concentrations (Obs–Pred) *R*^2^ = 0.78). No continuous covariates explored resulted in a clear improvement over the base model. Inclusion of anticoagulation modality and vasopressor use as categorical covariates resulted in similar PK parameter estimates, with a trend towards lower parameter estimate variability when using regional citrate anti-coagulation or without vasopressor use. Simulations using PTA plots suggested that a 2 g loading dose followed by 750 mg 12 hourly as maintenance dose, commencing 12 h after loading, is required to achieve adequate early target trough concentrations of at least 15 mg/L.

**Conclusions:**

PTA simulations suggest that acceptable trough vancomycin concentrations can be achieved early in treatment with a 2 g loading dose and maintenance dose of 750 mg 12 hourly for critically ill patients on CVVHDF.

**Supplementary Information:**

The online version contains supplementary material available at 10.1186/s13054-021-03863-4.

## Introduction

Multi-drug resistant pathogens in the ICU setting continue to increase. It is now more important than ever that antibiotics amenable to therapeutic drug monitoring (TDM) reach their targets consistently. This is particularly challenging in septic patients requiring renal replacement therapy [1]. The pharmacokinetics (PK) of critically ill patients are complex as they may have multiple co-existing chronic conditions in addition to their acute illness [2]. In individual patients, changes in PK parameters can occur over time as their illness trajectory changes [3, 4].

Continuous renal replacement therapy (CRRT) using regional citrate anticoagulation (RCA) is becoming standard practice. Antibiotic clearance using RCA may differ from CRRT using standard anticoagulation modalities given that it utilises a different dialysis prescription from that used for CRRT with or without heparin anticoagulation. RCA anticoagulation is also associated with prolonged dialysis “circuit life” and less bleeding compared to non-RCA anticoagulation [5]. Furthermore, there is likely to be inherent bias (i.e. patient types suitable for RCA) in RCA use. Thus the effect of RCA vs. non-RCA dialysis modalities on clearance (CL) is unknown.

Population PK modelling is a powerful tool for individual dose optimisation and to support dosing regimen selection in a specific population. It facilitates estimation of PK parameters for a population and identification of patient-related covariates for the estimated parameters [6]. For example, if renal function is identified as a covariate for drug clearance in a population PK model, a patient’s drug clearance estimate can be individualised based on their renal function. When an adequate population model has been identified to describe drug PK data in a population, this model can then be used to investigate the likely ability of different dosing regimens to achieve pharmacokinetic-pharmacodynamic (PKPD) targets. This is done through generation of probability of target attainment (PTA) plots. These plots are usually constructed through generation of thousands of simulated profiles using Monte Carlo simulation methods based on the original population PK model. The results of these simulated profiles are then used to calculate the probability of different dosing regimens attaining specified target concentrations.

In view of the challenges of antibiotic dosing in septic patients on renal replacement therapy and concerns around achieving optimal vancomycin concentrations in the context of increasing drug resistance, we undertook a PK analysis to support optimal therapeutic vancomycin dosing. Our aim was to develop a population PK model for intermittent infusion of vancomycin in critically ill patients receiving continuous venovenous haemodiafiltration (CVVHDF), to explore the influence of dialysis-related and selected patient covariates on the PK parameter estimates, and to use PTA plots to illustrate the trajectory of target vancomycin level attainment over time with different dosing regimens.

## Methods

### Study design, setting, patients and data collection

A retrospective study of vancomycin levels in patients on CVVHDF was performed in a single-centre tertiary level adult intensive care unit (ICU) on patient data recorded between 22nd January 2015 and July 2016. The study had local research ethics approval (St. James’s Hospital/Tallaght University Hospital Joint Research Ethics Committee Reference: 2016-04 List 13 (13), 2015-09 Vice-chairman Action (7), 2019-02 List 5 (15)). Data collection was from the Electronic Health Record (EHR), (Intellispace Critical Care and Anaesthesia (ICCA, Phillips^®^)).

The following data were collected from the EHR:

- Vancomycin infusion dose and start time
- Vancomycin concentration data
- Clinical, physiological (APACHE II and SOFA scores) and laboratory data
- Dialysis-related data.

Patients receiving vancomycin on CVVHDF were included if they had at least one peak and trough vancomycin level during one or more dose intervals, with CVVHDF in use for the majority of the dosing interval; Data were included from the longest period of consecutive dosage intervals for each patient. Patients commenced on vancomycin before admission to ICU were included only if a trough concentration was available prior to the first dosing interval on ICU used in the analysis.

### Vancomycin therapy and TDM

The ICU policy was to give patients vancomycin loading doses of 25 mg/kg, followed by once-daily maintenance doses 15–20 mg/kg with guidance from levels, delivered by infusion pump at a rate of 10 mg/min. A trough and peak serum concentration of 15–20 mg/L and 20–40 mg/L were targeted, respectively. Peak levels were taken 60 min after the end of infusion and trough levels were taken immediately before the next dose. The ‘Architect iVancomycin^®^’ assay used an in vitro chemiluminescent microparticle immunoassay to quantitatively measure serum vancomycin (mg/L).

### Continuous renal replacement therapy (CRRT)

CVVHDF was employed using a Prismaflex^®^ machine, with a polyarylethersulfone (PAES) haemofilter. Blood flow rate (*Q*_b_) ranged from 120 to 220 mL/min. Effluent flow rate (*Q*_eff_) was calculated using the formula: dialysate rate (*Q*_dial_) + replacement rate (*Q*_rep_) + actual fluid removal (measured ultrafiltration) rate + pre-blood pump (PBP) fluid rate. The anticoagulation systems were classified into RCA or non-RCA-based systems. Dosage intervals were categorised according to the dialysis category for the majority of the dosage interval; RCA or non-RCA. The non-RCA systems included either unfractionated heparin or no anticoagulation.

### Population pharmacokinetic analysis

As concentration data were limited to peak and trough data only, with peaks taken at the uniform time of 60 min after the end of the infusion, a one-compartment model based on clearance (CL) and volume of distribution (V) parameter estimates was developed using non-parametric adaptive grid algorithm in the population PK package for R, Pmetrics (http://www.lapk.org/pmetrics.php) [7].

#### Continuous covariates

Continuous covariates tested for inclusion in the model consisted of dialysis-related, laboratory and clinical data including *Q*_eff_, flux, haemofiltration component, *Q*_dial_, actual fluid removal, *Q*_b_, absolute cumulative balance, difference in absolute cumulative balance, body weight, albumin concentration, C-reactive protein concentration, capillary leak index, white cell count, noradrenaline dose and body weight. Covariates were normalised relative to the population covariate median, and focussed on variation of covariate with CL, apart from body weight which was also investigated with V. Model performance was assessed using linear and exponential relationships, with sample equation formats in Additional file 1: Table S1.

#### Categorical covariates

Categorical covariates with CL were also tested for inclusion in the model. Each dosing interval was categorised using binary categorisation: RCA or non-RCA, fluid balance change between dosage intervals (accumulation vs. loss) and presence/absence of vasopressor.

Final model selection was based on visual observation of the plots of observed versus predicted concentrations and the associated coefficient of determination of the linear regression of the observed versus predicted concentrations (*R*^2^ values), bias, imprecision, and reduction in twice the log likelihood (− 2 * LL), Akaike Information Criterion (AIC) and Bayesian Information Criterion (BIC). A significant (*p* < 0.05) improvement in model fit was determined by comparing the base and covariate models considering the reduction of − 2 * LL against a *χ*^2^ distribution with consideration of degrees of freedom (CI 95%). Of note, emphasis was placed on the population prediction model rather than individual posterior predictions, in order to identify a model which would facilitate application of PK-covariate relationships to initial dosing in patients from a similar population. Internal model validation was conducted through generation and inspection of residual and visual predictive plots.

### Probability of target attainment (PTA) plots

PTA plots were generated by performing Monte Carlo simulations (*n* = 1000) using the final (base) model. Four exploratory dosing regimens were considered for determination of initial therapeutic vancomycin dosing: 2 g loading dose followed by either 750 mg or 500 mg every 12 h, 2 g loading followed by 1.5 g at 12 h and then 1.5 g 24-hourly thereafter, or 1.5 g loading followed by 750 mg every 12 h. The investigated target was trough concentration every 12–24 h as relevant up to 48 h post-first-dose. Our unit vancomycin trough target range is 15–20 mg/L. We considered at least an 80% probability of achieving a trough of 15 mg/L from 12 h onwards to be clinically acceptable. To consider potentially toxic peak concentrations, PTA plots for peak concentrations of 20–40 mg/L after the 36 h dose were generated.

As an additional sensitivity analysis, we produced PTA plots for AUC/MIC (area under the curve/minimum inhibitory concentration) of vancomycin at 24–48 h treatment, given the recent change in guidelines which now recommends AUC-guided dosing and monitoring of vancomycin, though not yet adopted in our clinical practice [8].

## Results

### Patient characteristics

A total of 106 vancomycin dosing intervals in 24 patients were included in the final analysis; 74 peak and 96 trough levels were studied. Table 1 shows the main demographic and clinical characteristics along with vancomycin dose and concentration data in Table 2. Of note, the average and median vancomycin trough levels are at the lower end of the target range.

**Table 1:** Patient demographics

Patient demographics *n* = 24	*n*/Total or mean (SD)	Median (IQR)
Male, *n* (%)	18/24 (75%)	
Age (years)	65.5 (12.3)	67 (56.8–75.3)
Weight (kg)	81.8 (24)	79.9 (61.3–91.5)
BMI (kg/m^2^)	28.6 (8.2)	26.8 (22.8–31)
ICU length of stay (days)	17.4 (19)	12 (6.2–20.9)
APACHE score	23 (5)	23 (20–27)
SOFA score	10.5 (3.2)	11 (8–12.5)
Urine output/24 h on day 1 of study (mL)	238.6 (344.2)	77.9 (11.8–333.3)
In-hospital mortality, *n* (%)	6/24 (25%)	

	Range	Median (IQR)
Urine output/24 h during study period (ml)	0–1836	2.5 (0–92.5)

IQR, Interquartile range; BMI, body mass index; ICU, intensive care unit; APACHE, acute physiologic assessment and chronic health evaluation; SOFA, sequential organ failure assessment

**Table 2:** Vancomycin administration results

Vancomycin administration	Number of dose intervals included	Mean (SD)	Median (IQR)
Total number of vancomycin dosage intervals^a^	106	4.46 (2.7)^b^	4 (2–6)^b^
Vancomycin dose (mg)	107	1098 (249.4)	1000 (1000–1250)
Vancomycin serum peak TDM level (mg/L)	74	29.1 (7.5)	26.5 (24.5–33.3)
Vancomycin serum trough TDM level (mg/L)	96	15.7 (5.1)	15.2 (12–18.6)
Length of dosage interval (h)	106	18.5 (7)	18.2 (12.3–23.8)

IQR, Interquartile range; TDM, therapeutic drug monitoring

^a^Only dose intervals that met the inclusion criteria were included

^b^Mean and median number of vancomycin dosage intervals per patient

### Population pharmacokinetic analysis

#### Base structural model (no covariates)

**Fig. 1:**
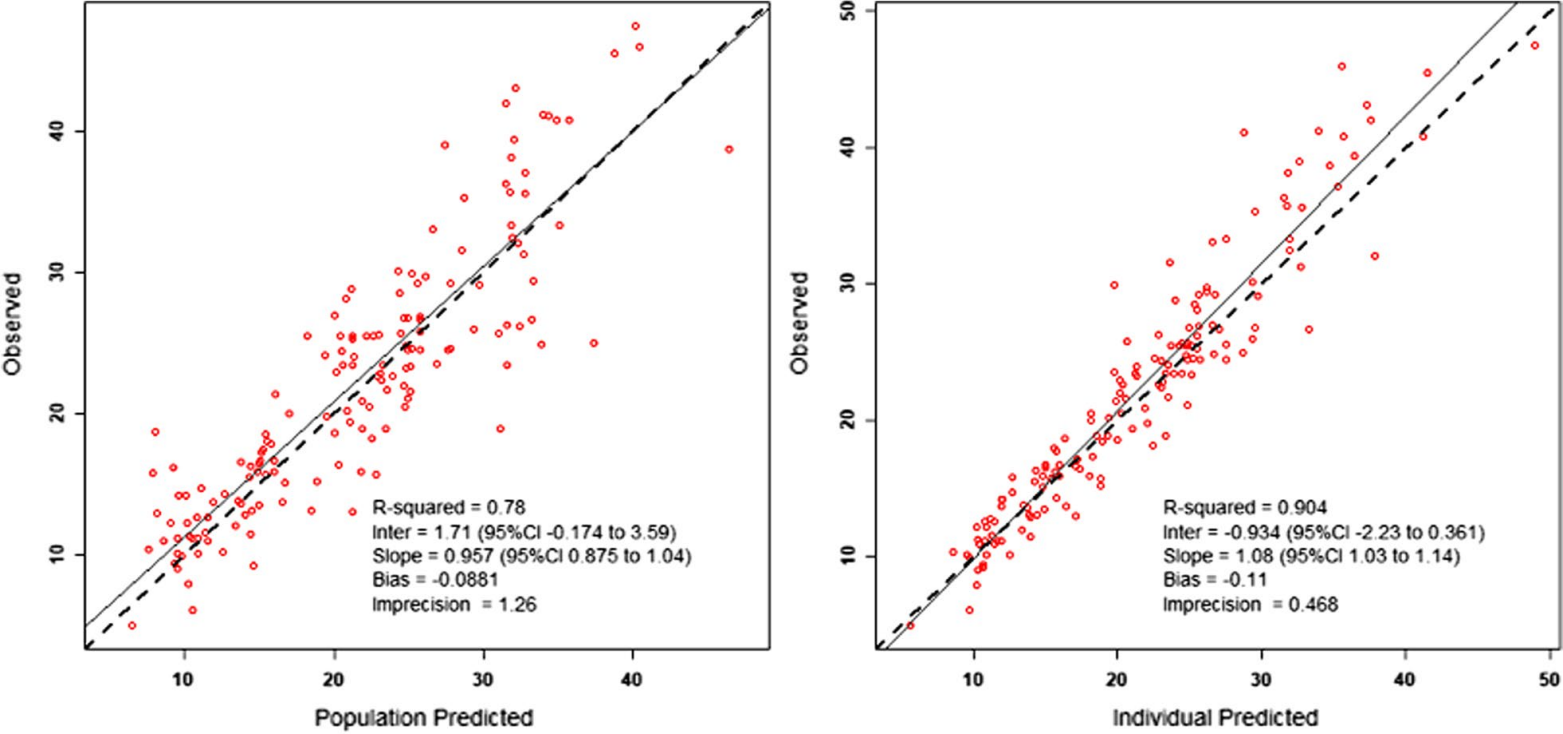
Diagnostic observed versus predicted concentration plots from the structural base model. The left panel describes the population predicted plot. The right panel describes the posterior individual predicted plot

In the base model, the mean CL was 2.59 L/h ± 0.49 with a mean V of 80.98 L ± 16.89; ~ 1L/k g based on mean body weight of our study cohort (Table 3). Diagnostic population predicted versus observed data showed strong predictive ability of the base model with an *R*^2^ of 0.78 (Fig. 1).

**Table 3:** PK parameter estimates for the structural base model, the categorical model which includes the effect of citrate (RCA) versus non-citrate (non-RCA) systems, and presence of vasopressor; and sample models including continuous dialysis covariates

	Parameter	Mean	SD	CV%	Median
*Base CL model*					
Base model	CL (L h^−1^)	2.59	0.49	18.99	2.70
	*V* (L)	80.98	16.89	20.86	73.72
*Model based on categorical (RCA or non-RCA) covariate (CL1 reflects intervals on RCA)*					
Category applied to CL	CL1 (L h^−1^)	2.73	0.25	9.14	2.58
	CL2 (L h^−1^)	2.54	0.57	22.53	2.50
	*V* (L)	81.13	15.83	19.51	73.38
*Model based on categorical (vasopressor presence) covariate (CL1 reflects intervals with vasopressor use)*					
Category applied to CL	CL1 (L h^−1^) CL2 (L h^−1^)	2.73	0.72	26.48	2.77
		2.53	0.33	12.97	2.53
	*V* (L)	77.94	12.44	15.96	75.25
*Sample models including continuous dialysis covariates*					
*Q*_eff_ applied to CL, linear model	CL1 (L h^−1^) CL2 (L h^−1^)	1.606 0.89	1.1 0.74	68.33 83.1	1.42 0.82
	*V* (L)	79.69	12.13	15.22	76.17
Flux applied to CL, exponential model	CL1 (L h^−1^) CL2 (L h^−1^)	1.88 0.36	0.83 0.33	44.43 92.22	2.12 0.23
	*V* (L)	79.62	12.31	15.47	73.99

Although the model based on continuous dialysis covariates resulted in significant differences in −2LL, there was little improvement in other model comparison metrics (see Additional file 1: Table S2), a higher CV% than base model parameter estimates and a high (> 0.9) correlation between CL1 and CL2 estimated values; prompting caution in their interpretation. CL1 and CL2 represent RCA and non-RCA, or vasopressor and non-vasopressor use, respectively, in the categorical covariates added to the base model. For continuous covariate models, CL1 and CL2 represent the parameters as presented in the sample equations in Additional file 1: Table S1; For the linear model CL1 is the intercept and CL2 is the coefficient associated with change in covariate relative to median value, for the exponential model CL1 is the coefficient and CL2 is the constant part of the exponent which varies with the change in covariate relative to the median value

CL, Clearance; CV, coefficient of variability (CV = SD/mean); *Q*_eff_, effluent flow rate; SD, standard deviation; V, volume of distribution

#### Continuous covariates

Although multiple covariate models resulted in a statistically significant improvement over the base model in terms of reduction of − 2LL, these represented no, or very minimal, improvement in other model comparison metrics and therefore their clinical significance was difficult to determine. These covariates included most dialysis-related covariates such as *Q*_eff_, flux, haemofiltration component, *Q*_dial_ and actual fluid removal, in addition to functions of noradrenaline dose and actual fluid balance. Examples of parameter estimates from these models are presented in Table 3. Of note, in the linear model, the estimate of the CL component associated with variation in *Q*_eff_ is small and variable, and the variability (CV%) around parameter estimates in the dialysis models is higher overall than the base model estimates. Furthermore, there was a high correlation (> 0.9) between CL1 and CL2 estimates in the models using dialysis covariates, prompting caution in the interpretation of the specific parameter estimates. Interestingly, inclusion of body weight as a covariate with V did not result in a statistically significant improvement to the base model in this cohort.

#### Categorical covariates

The presence of vasopressor as a categorical covariate resulted in a statistically significant improvement in model predictability, however, there was no practical difference between the PK estimates for the base model and the models where CL was categorised by either dialysis anticoagulation modality or vasopressor presence (Table 3). The coefficients of variation (CV%) for the CL estimates were 9.14% and 22.53% in the RCA and non-RCA group, and 26.48% and 12.97% for presence/absence of vasopressor, respectively (Table 3).

No single covariate model provided a notable improvement simultaneously in both population predictions and posterior individual predictions. Given that the base structural model adequately described the data and the lack of any clear model improvement on covariate inclusion, the base structural model was used for generation of the PTA plots.

### Probability of target attainment plots

**Fig. 2:**
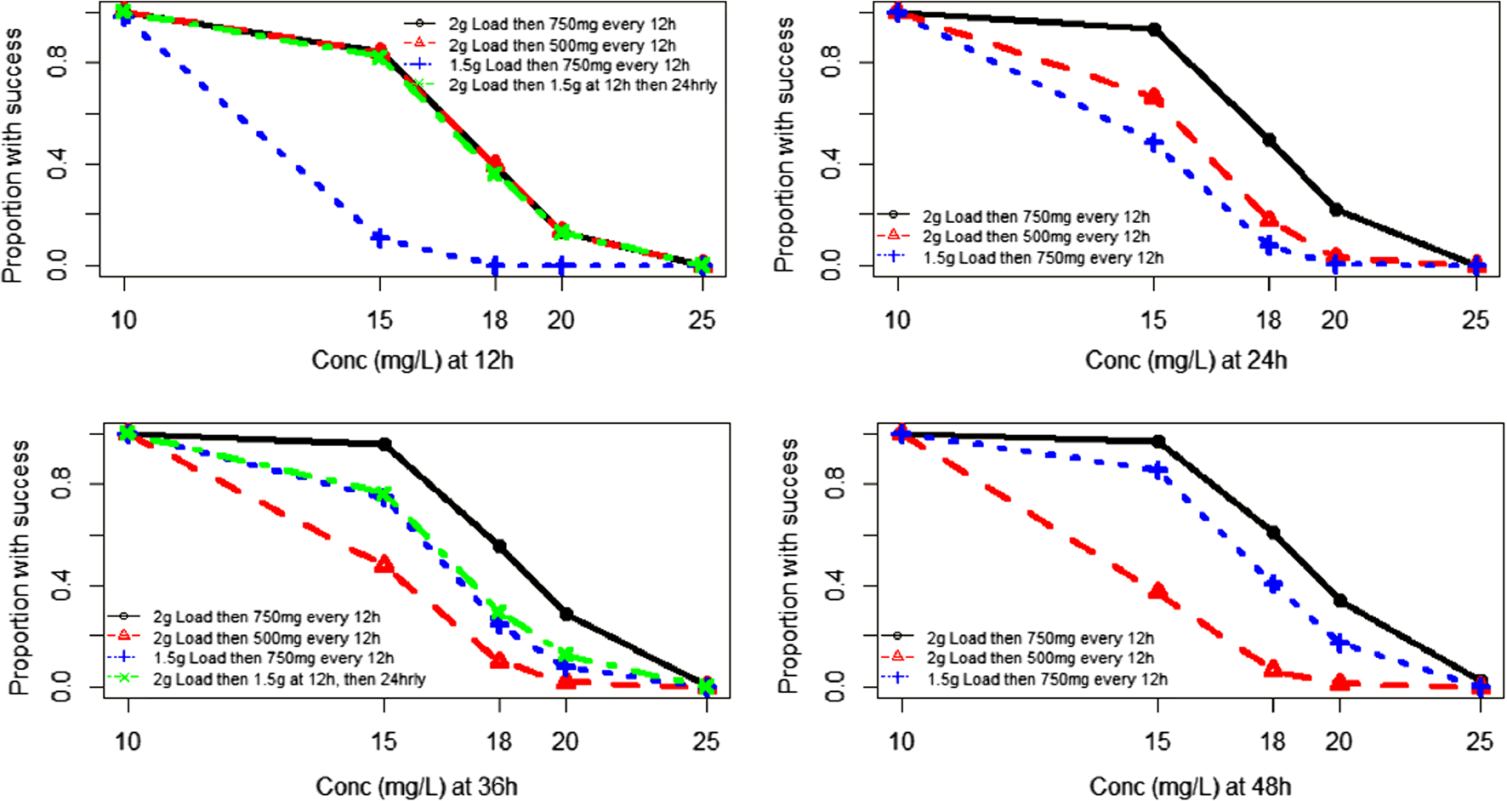
PTA plots illustrating the proportion of simulated patient concentrations attaining success at each concentration. Success is defined as attaining the concentration detailed on the horizontal axis. In each case, simulated concentrations are immediately pre-dose

The PTA plots are presented in Fig. 2. Plots were generated for 48 h post-first dose as it is considered that after this time dosing would be guided by TDM.

For Regimen 1 (2 g loading then 750 mg every 12 h), the approximate probability of a level of 15 mg/L was > 80% at 12 h, > 90% at 24, 36 and 48 h. The approximate probability of a level of 20 mg/L was < 20% at 12 h, 20–30% at 24 h and 36 h and 40% at 48 h.

For Regimen 2 (2 g loading then 500 mg every 12 h), the approximate probability of a level of 15 mg/L was > 80% at 12 h, 70–80% at 24 h, 50% at 36 h and 40% at 48 h. The approximate probability of a level of 20 mg/L was < 20% at 12 h, and < 10% at 24, 36 and 48 h.

For Regimen 3 (1.5 g loading then 750 mg every 12 h) the approximate probability of a level of 15 mg/L was 10–20% at 12 h, 50% at 24 h, 70% at 36 h and > 80% at 48 h. The approximate probability of a level of 20 mg/L was negligible at 12 and 24 h, and 10% at 36 h and 20% at 48 h.

For Regimen 4 (2 g loading, then 1.5 g at 12 h, then 24 hourly), the approximate probability of a level of 15 mg/L was > 80% at 12 h and 70–80% at 36 h. The approximate probability of a level of 20 mg/L < 20% at 12 and 36 h. Plots at 24 and 48 h were not applicable due to the dosing regimen.

The probability of a level of 25 mg/L was negligible at all investigated time points for each of the simulated dosage regimens.

As target vancomycin trough concentrations are 15–20 mg/L, the results suggest that a 2 g loading dose would be required to achieve adequate trough concentrations (> 15 mg/L) early in treatment. A maintenance dose of 750 mg 12 hourly results in a high probability of achieving at least 15 mg/L levels with approximately 20–30% of patients achieving a level of 20 mg/L and a very small percentage levels > 20 mg/L.

Additional file 1: Fig. S1 gives PTA plots for AUC/MIC of vancomycin at 24–48 h of treatment. This aligned with our advocated dose of 2 g vancomycin loading, followed by 750 mg 12 hourly, which resulted approximately in a 100% probability of attaining a target AUC of 400 mg/L * h for MIC 1 mg/L, in keeping with the recommended target AUC/MIC ratio for clinical efficacy from the guidelines [8]. Additional file 1: Fig. S2 gives PTA plots for peak concentration after the 36 h dose, based on the timepoint (h) closest to when the peak would be measured in practice for each infusion. To mitigate risks of toxicity, it is recommended to avoid peaks in excess of 40 mg/L, and the simulated dosage regimens explored in the current work are not associated with a risk of toxic peak concentrations.

## Discussion

In this single-centre, retrospective population pharmacokinetic analysis of PK of vancomyin following intermittent infusion in critically ill patients receiving CVVHDF, PTA plots suggest that we could achieve acceptable trough concentrations early in treatment with a 2 g loading dose and maintenance dose of 750 mg 12 hourly for most patients. No continuous covariates explored resulted in a clinically significant improvement over the base pharmacokinetic model. Selection of vancomycin loading doses using a population PK model has been shown to result in better attainment of therapeutic concentrations than standard loading doses in critically ill patients, excluding those on hamodialysis [9]. The results of the current study present an opportunity to employ a population model to inform dose selection in patients on CVVHDF.

We found that the predictive ability of the base structural pharmacokinetic model was higher than expected. This was possibly due to the strict inclusion criteria which used only dosing intervals where CVVHDF downtime was less than time on CVVHDF. Given the good predictive ability of the base model it was difficult to identify covariates in this current data set which resulted in a notable increase in model predictive ability. This finding shows that the detail available from EHR data can aid development of PK models representing more specific patient cohorts and clinical scenarios. Despite the lack of effluent data pertaining to drug removal via CVVHDF, our analyses illustrate the potential to identify relevant dialysis-related covariates for total drug CL from routine clinical information retrieved from the clinical information system. A statistically significant improvement was identified in several models incorporating dialysis-related continuous covariates, when they were explored individually. *Q*_eff_ and *Q*_b_ can be considered determinants of dialysis dose and it could be anticipated that the combination of these covariates might represent a covariate for CL. In the current work, the reduction in −2LL in the model with *Q*_b_ as a CL covariate was minimal, and in fact there was a slight deterioration in predictive ability from goodness-of-fit plots (Additional file 1: Table S2). Possibly a difference in *Q*_b_ prescription in the citrate versus non-citrate cohorts may have confounded this result, or the contribution of *Q*_b_ may be less notable than that of *Q*_eff_. The lack of improvement of the model including *Q*_b_ as a covariate over base model therefore did not warrant its investigation in combination with *Q*_eff_, however investigation of this combination in a single dialysis modality cohort may be more fruitful. The current work also resulted in a statistical improvement where there was inclusion of vasopressor use as a categorical covariate. Ultimately, the presence of these small significant effects suggests that these covariates warrant further prospective investigation with richer data. Such an investigation would also support better characterisation of distribution effects, which would facilitate more detailed exploration of albumin concentration, capillary leak index and fluid balance metrics as covariates.

The PK parameter estimates in the current model align well with estimates from the literature. Previous work by Okada et al. [10] examined the vancomycin PK in five patients with multiple organ failure who received CVVHDF and found that the mean total vancomycin CL was 1.7 L/h (29 ± 1.8 mL/min) and the mean V was 54.4 ± 10.2 L (1.21 ± 0.26 L/kg). Udy et al. reported that the median total vancomycin CL was 2.9 L/h (IQR 2.4–3.4 L/h) and the median V was 0.8 L/kg (IQR 0.6–1.1 L/kg) in a large retrospective study of patients receiving either continuous venovenous haemofiltration (CVVH) or CVVHDF using a one-compartment model [11]. Similarly, Roberts et al*.* reported a mean vancomycin clearance of 22 mL/min versus 28 mL/min for standard intensity versus higher intensity continuous renal replacement therapy [12]. The small number of dosing intervals on RCA in the current study and the suggestion of more variability around the CL estimate in the non-RCA group suggest that characterisation of vancomycin PK with respect to RCA use is worthy of further consideration, enabling identification of subgroups potentially requiring more focused TDM.

As target concentrations are 15–20 mg/L, our PTA plots suggest that a 2 g loading dose would be required to achieve adequate trough concentrations (> 15 mg/L) early in treatment. The unit policy of a 25 mg/kg loading dose would result in a patient of average body weight in this cohort (82 kg) receiving a dose of approximately 2 g. This suggests that patients below average body weight might be inadequately loaded. Although there may be clinical reticence to prescribe substantial vancomycin doses to patients with acute kidney injury (AKI) on CRRT, the average trough concentration of 15.7 mg/L noted in Table 2 suggests that there is scope to revisit the loading doses used in this cohort. Given that many patients receive the first vancomycin dose prior to entering ICU, with 18/24 (75%) patients in our study commencing vancomycin treatment prior to CVVHDF, these results suggest a need to focus on adequate loading doses to ensure therapeutic concentrations are achieved. The PTA plots suggest that a maintenance dose of 750 mg commencing 12 h post dose results in a high probability of achieving at least 15 mg/L pre-dose levels but is also associated with a higher probability of achieving potentially undesirably high concentrations, with approximately 20–30% of patients predicted to achieve concentrations of 20 mg/L. Notwithstanding this, the proportion of patients with predicted concentrations of 25 mg/L is low when using this high dose. Use of a 500 mg 12 hourly maintenance dose could be considered in instances where there is a toxicity concern; though the model predicts decreasing proportions will have successful concentrations with this maintenance dose from 12 to 48 h, despite the initial loading dose.

Recent guidelines suggest that AUC/MIC ratios should be used rather than trough measurements for therapeutic dose monitoring of vancomycin [8]. The evidence base for use of target AUC/MIC ratios on CRRT is not as strong but is still recommended at present. Given that a Bayesian dose adjustment is advocated for AUC-based TDM, using a PK model to inform the initial dose, we suggest that developing population PK models for initial vancomycin dosing in the CRRT population is a step towards facilitating AUC-based TDM in clinical practice.

The strengths of our study include use of detailed clinical, biochemical and machine data taken from the electronic health record. This allowed us to explore pharmacokinetic models which incorporated individual patient and CRRT therapy characteristics. Our use of PTA plots provided a method for informing clinical dosing in this patient cohort. There was little CRRT downtime during our vancomycin dosing intervals which allowed for a consistent assessment of the impact of CRRT on vancomycin clearance.

Study limitations include that this was a single-centre study with retrospective data collection, and prospective model validation is advised. The availability of peak and trough levels alone restricted our analysis to a one-compartment model. Unequal numbers of samples across patients with small patient numbers in a single-centre limited the data available for analysis. No comparative data were available for non-renal CL of vancomycin, nor the unbound/free vancomycin concentrations, which can affect PK parameters [13].

## Conclusion

In conclusion, our single-centre, retrospective population analysis of PK of vancomyin following intermittent infusion in critically ill patients receiving CVVHDF suggests that, based on PTA plots, we could achieve acceptable trough concentrations early in treatment with a 2 g loading dose and maintenance dose of 750 mg 12 hourly for the majority of patients. No continuous covariates explored resulted in a clinically significant improvement over the base pharmacokinetic model. Vasopressor use, RCA status and dialysis-related covariates are suggested for further analysis with richer data, to optimise covariate PK modelling of vancomycin in ICU patients on CVVHDF and support dose optimisation. It is crucial that an adequate vancomycin loading dose is administered early in therapy and followed by a suitable maintenance regimen to sustain adequate vancomycin levels.

## Supplementary Information

**Additional file 1: Supplemental Table S1:** Format of covariate analyses. **Supplemental Table S2:** Model comparison metrics from the base model, examples of “best” models (Qeff and flux as clearance covariates) and from model inclusion of Qb as covariate for clearance. **Supplemental Figure S1:** PTA plot for AUC of vancomycin at 24-48 hours of treatment. **Supplemental Figure S2:** PTA plots for peak vancomycin levels relating to the 36h dose, at A) 38h for the regimens with 500 mg and 750 mg 36h dose and B) at 39h for the regimen with 1500 mg given at 36h. “Success” reflects attainment of the relevant peak concentration at 38h, in this case considered to be a metric of toxicity if > 40 mg/L.

## Data Availability

The datasets generated and analysed during this study are not publicly available given concerns for patient confidentiality as this is a single-centre study but are available from the corresponding author on reasonable request.

## Abbreviations

AKI: Acute kidney injury
AUC/MIC: Area under the curve/minimum inhibitory concentration
BIC: Bayesian information criterion
CL: Clearance
CVVH: Continuous venovenous haemofiltration
CVVHDF: Continuous venovenous haemodiafiltration
EHR: Electronic health record
ICU: Intensive care unit
PAES: Polyarylethersulfone
PBP: Pre-blood pump
PK: Pharmacokinetics
PKPD: Pharmacokinetic-pharmacodynamic
PTA: Probability of target attainment
*Q*_b_: Blood flow rate
*Q*_dial_: Dialysate flow rate
*Q*_eff_: Effluent flow rate
*Q*_rep_: Replacement rate
RCA: Regional citrate anti-coagulation
TDM: Therapeutic drug monitoring
*V*: Volume of distribution
