# Supplementary material for: Exploring population pharmacokinetic models in patients treated with vancomycin during continuous venovenous haemodiafiltration (CVVHDF)
Source: Crit Care. 2021 Dec 20;25:443. doi: 10.1186/s13054-021-03863-4 (PMC8691013; doi:10.1186/s13054-021-03863-4)
Supplement: Supplementary file 1 — Additional file 1: Supplemental Table S1: Format of covariate analyses. Supplemental Table S2: Model comparison metrics from the base model, examples of “best” models (Qeff and flux as clearance covariates) and from model inclusion of Qb as covariate for clearance. Supplemental Figure S1: PTA plot for AUC of vancomycin at 24-48 hours of treatment. Supplemental Figure S2: PTA plots for peak vancomycin levels relating to the 36h dose, at A) 38h for the regimens with 500 mg and 750 mg 36h dose and B) at 39h for the regimen with 1500 mg given at 36h. “Success” reflects attainment of the relevant peak concentration at 38h, in this case considered to be a metric of toxicity if > 40 mg/L. [file 13054_2021_3863_MOESM1_ESM.docx]

Supplemental Table S1: Format of covariate analyses

| 1. **Covariate relationship** | **Formula used to express covariate inclusion in the model** |
| --- | --- |
|  |  |
| Linear with intercept | $CL=CL1+CL2*\frac{COVARIATE}{MEDIAN}$ |
| Exponential | $CL=CL1*EXP(CL2*\frac{COVARIATE}{MEDIAN})$ |
|  |  |
| Categorical* | $CL=CL1*COVARIATE+CL2*(1-COVARIATE)$ |

*Categorical data used value of ‘1’ or ‘0’ for the binary covariate categorisation. Abbreviations: CL, Clearance

**Supplemental Table S2: Model comparison metrics from the base model, examples of “best” models (Qeff and Flux as clearance covariates) and from model inclusion of Qb as covariate for clearance.**

| **Model** |  | | | **Population** | | | | **Individual** | | | |
| --- | --- | --- | --- | --- | --- | --- | --- | --- | --- | --- | --- |
|  | -2LL | AIC | BIC | R^2^ | Bias | Imp | Slope | R^2^ | Bias | Imp | Slope |
| **Base** | 796 | 802 | 811 | 0.78 | -0.09 | 1.26 | 0.957 | 0.904 | -0.11 | 0.47 | 1.08 |
| **Qeff -linear model** | 774 | 782 | 794 | 0.80 | 0.57 | 1.9 | 0.956 | 0.913 | -0.10 | 0.57 | 1.08 |
| **Flux-exponential model** | 774 | 782 | 794 | 0.8 | 0.59 | 1.87 | 0.928 | 0.915 | -0.09 | 0.56 | 1.07 |
| **Qb-Linear** | 793 | 801 | 813 | 0.77 | -0.17 | 1.36 | 0.952 | 0.903 | -0.08 | 0.48 | 1.08 |

*Abbreviations: Qeff: Effluent Flow Rate; Qb-Blood Flow Rate; -2LL: -twice the log likelihood; AIC: Akaike Information Criterion; BIC: Bayesian Information Criterion; Imp: Imprecision*

**Supplemental Figure S1: PTA plot for AUC of vancomycin at 24-48 hours of treatment**

**Abbreviations: AUC, area under the curve**

**Supplemental Figure S2: PTA plots for peak vancomycin levels relating to the 36h dose, at A) 38h for the regimens with 500 mg and 750 mg 36h dose and B) at 39h for the regimen with 1500 mg given at 36h. “Success” reflects attainment of the relevant peak concentration at 38h, in this case considered to be a metric of toxicity if > 40 mg/L**

**A)**

**B)**
